# Coping with the Conflict-of-Interest Pandemic by Listening to and Doubting Everyone, Including Yourself

**DOI:** 10.1007/s11948-015-9658-9

**Published:** 2015-05-30

**Authors:** Lynn T. Kozlowski

**Affiliations:** Department of Community Health and Health Behavior, School of Public Health and Health Professions, University at Buffalo, State University of New York, 323 Kimball Tower, 3435 Main Street, Buffalo, NY 14214-8028 USA

**Keywords:** Conflict of interest, Industry funding, Ethics, Peer-review, Bias in research, Responsible conduct of research

## Abstract

In light of the widespread existence of financial and non-financial issues that contribute to the appearance or fact of conflict of interest, it is proposed that conflict of interest should generally be assumed, no matter the source of financial support or the expressed declarations of conflicts and even with respect to one’s own work. No new model is advanced for modification of peer-review processes or for elaboration of author declarations of interest. Researchers should be assessing the quality of published work as best they can and make their own decisions on the appropriate use of the work. While some apparent sources of conflict are likely more obvious and serious than others, even subtler biases can influence scientific reports. Ignoring peer-reviewed contributions because of conflict-of-interest concerns is discouraged. Listening skeptically to all sources, including yourself, is encouraged.

## Introduction

Conflict of interest was somewhat easier to deal with [but not easy, e.g. (Roseman et al. 2011)] when the focus was on finances. Exploring the ecology of bias and pursuing it down into the complex psychology of the individual with impaired rational judgment (Cain and Detsky 2008), abiding “moral psychological” prejudices (Haidt 2007), and needs for self-affirmation (Cohen and Sherman 2014), one can become hopeless about controlling the appearance or fact of conflict as an ingredient in all of our scientific products (Abdoul et al. 2012; Cain and Detsky 2008; Lampe 2012; The PloS Medicine Editors 2008). Richard Feynman may have captured the personal challenges to scientific integrity most succinctly: “The first principle is that you must not fool yourself–and you are the easiest person to fool. So you have to be very careful about that. After you’ve not fooled yourself, it’s easy not to fool other scientists. You just have to be honest in a conventional way after that” (Feynman 1985). Not fooling yourself remains an abiding challenge.

## Playing on the Fields of Peer-Reviewed Research

Publishing peer-reviewed research, especially in higher-profile journals, often requires advocacy and argument to win through to publication. On scholarly issues, there will be “winners” who are proven right by findings and “losers” who drop in credibility and influence. Modern science is often a team sport with “star” teams and “star” players who savor and benefit from stardom (cf. Latour 1987). Perhaps applied sciences should “operate with an assumption of bias, with the onus of proof on applied medical scientists to facilitate the ‘data transparency’ necessary to validate their research” (Roseman et al. 2011).

Many journals now provide options for prospective authors to exclude certain reviewers as conflicted, and this can influence the likelihood of acceptance (Goldsmith et al. 2006) as can the recommendation of preferred reviewers (Schroter et al. 2006). When the battle lines have been drawn on a controversial issue, knowledgeable editors can be challenged to decide who should be fairly selected to perform reviews because they are likely to be aware that choice of one reviewer is likelier to lead to rejection while the choice of another would likelier lead to acceptance. Unavoidably, “party lines” can start to develop in certain publications, such that they can be viewed by authors to be more welcoming to articles holding certain positions. Such forces contribute to the evolution of new journals, to the extent that certain viewpoints can get closed out of publication in the existing journals. The proliferation of peer-reviewed journals further complicates the credibility of what makes it though peer-review. (On some days in past months I assume that I am not alone in having received two or three emails for new journals seeking editors and editorial board members.) Most of us will also have examples of peer-reviewed papers in prestigious journals that we ourselves would not have supported for publication anywhere.

Not all issues are laden with controversy, but in the area of public health, issues like needle exchange programs, sex education, mandatory vaccinations, and harm reduction in tobacco use are all charged topics that engage moral psychological values as well as scientific values (Alderman et al. 2010; Kozlowski 2013, 2015). If one’s research can have little effect on policy, profits, disability, or death, then, it may trigger less controversy; however, one should not underestimate the ego-involvement in defense of any intellectual offering.

## The About-Face Test

H.L. Mencken, the noted social critic, offered a rule-of thumb for assessing conflict-of-interest (Mencken 1923):

When I encounter a new idea, whether aesthetic, political, theological, or epistemological, I ask myself, instantly and automatically, what would happen to its proponent if he should state its exact antithesis. If nothing would happen to him, than I am willing and eager to listen to him. But if he would lose anything valuable by a *volte face* – if stating his idea is profitable to him, if the act secures his roof, butters his parsnips, gets him a tip – then I hear him with one ear only. He is not a free man.

*Volte face* refers to one doing an about-face or making a U-turn, to reverse an opinion by 180°. Mencken’s account provides a useful broad metaphor for conflict of interest (not being free to take an opposing position), and the more we understand the complex sources of bias, the clearer it is that this is an intrinsic problem (Fanelli 2015; Ioannidis 2005; Ioannidis et al. 2014; McNutt 2014; Ware and Munafò 2015). The science of controversial issues is rife with individuals (and organizations) (Kozlowski 2015) who, as Mencken says, should be listened to “with only one ear.” These individuals are in effect not free (completely free) to adopt opinions that would be unsupportive of the opinions of their agencies, superiors, close colleagues or even their own previous positions.

## Listening with One Ear—to Everyone

Peer-review has a long history, is still under-going change, and efforts to improve the process may have had limited success (Lee et al. 2013). Elaboration of author declarations of interest has been taking place, but may even be counter-productive (Loewenstein et al. 2012). I would encourage editors to avoid seeking unanimous opinions for offering publication. I also suggest that reviews by conflicted reviewers may at times be the most informed and valuable (Lee et al. 2013). Beyond that, I offer no new model for the methodology of peer-review, but I do encourage skepticism as well as direct evaluation by interested researchers of the quality of all peer-reviewed work. *Listening* to everyone, of course, does not mean *believing* everyone’s findings equally. One could propose a rating of conflicts according to their seriousness, and no doubt some sources of conflict merit heavy discounting (e.g., funding source and employer), but it is not clear that the most obvious or measurable conflicts are always the most serious. A “white hat bias” has been observed in which information becomes distorted “when in the service of what may be perceived to be righteous ends” (Cope and Allison 2010). Not fooling oneself may, in the end, be the greatest challenge and the hardest to measure.

What are we to do? Perhaps the best we can do is what Mencken did—listen with only one ear to views that are likely influenced by research party lines and institutional perspectives on the preferred questions, methods, and answers. Rather than developing an About-Face test to apply to reviewers and authors, I think the lesson of the psychology of bias is *listen to all voices with one ear*. So, when faced with industry-funded/conducted research on an important matter, whether the tobacco industry (which has been exiled from a number of journals) (Smith 2013), the pharmaceutical industry (which has generally not been excluded from any journal) (Elliott 2005; Smith 2013; Lexchin et al. 2003; Lexchin 2012; Washington 2011), or another business; or whether the reputation of the authors is at stake or anything that makes one suspicious that an about-face would be unwelcome, I encourage listening with one ear which has the advantage of still listening rather than ignoring or discounting completely. Since we too are subject to fooling ourselves, we should work to consider doubts about our own positions.

Systematic, critical reviews of literatures (e.g., *Cochrane Database of Systematic Reviews*, such as Singh et al. 2012) are important and helpful, but they too can be compromised by the quality of the work and biases lurking within publications being reviewed. Nevertheless, these reviews across several studies may provide better foundations for reports to the public or policymakers. Neither the public nor policymakers can be expected to assess the quality of research in the manner aspired to for advanced researchers. On certain topics like the health effects of diet, the public may have stopped listening to scientific reports because of the instability of the results (Nagler 2014). When only one interested funder is able to support certain research questions (Lexchin 2012), skeptical listening should certainly be emphasized, especially when the findings are not against interests. The exile of certain funders from peer-reviewed journals could prevent the identification of reliable, *conflicting* patterns of findings arising from different factions (e.g., Vartanian et al. 2007); such patterns can themselves be informative.

The point is to not fully discount anyone, but to be skeptical of everyone when listening carefully to all the reports one can find. Be wary of “arguments from authority” that may encourage belief in reports from the best journals and also be wary to not dismiss out-of-hand reports because of their funders. If conflict of interest is endemic and goes beyond finances to more challenging issues, it is probably best to listen to all voices, even our own, skeptically. The diversity of methods, approaches and biases may in the end lead to more robust conclusions and find things that a single perspectives will not. So, listen to everybody, but listen to everybody with one ear. The advantage of doubting yourself as well might even contribute to a change of position rather than to digging in deeper to defend what you have said before.
